# Multi-level analysis of electronic health record adoption by health care professionals: A study protocol

**DOI:** 10.1186/1748-5908-5-30

**Published:** 2010-04-23

**Authors:** Marie-Pierre Gagnon, Mathieu Ouimet, Gaston Godin, Michel Rousseau, Michel Labrecque, Yvan Leduc, Anis Ben Abdeljelil

**Affiliations:** 1 Research Center of the Centre Hospitalier Universitaire de Québec, Québec, Canada; 2 Faculty of Nursing Sciences, Université Laval, Québec, Canada; 3 Department of Political Science, Université Laval, Québec, Canada; 4 Department of Family Medicine, Faculty of Medicine, Université Laval, Québec, Canada

## Abstract

**Background:**

The electronic health record (EHR) is an important application of information and communication technologies to the healthcare sector. EHR implementation is expected to produce benefits for patients, professionals, organisations, and the population as a whole. These benefits cannot be achieved without the adoption of EHR by healthcare professionals. Nevertheless, the influence of individual and organisational factors in determining EHR adoption is still unclear. This study aims to assess the unique contribution of individual and organisational factors on EHR adoption in healthcare settings, as well as possible interrelations between these factors.

**Methods:**

A prospective study will be conducted. A stratified random sampling method will be used to select 50 healthcare organisations in the Quebec City Health Region (Canada). At the individual level, a sample of 15 to 30 health professionals will be chosen within each organisation depending on its size. A semi-structured questionnaire will be administered to two key informants in each organisation to collect organisational data. A composite adoption score of EHR adoption will be developed based on a Delphi process and will be used as the outcome variable. Twelve to eighteen months after the first contact, depending on the pace of EHR implementation, key informants and clinicians will be contacted once again to monitor the evolution of EHR adoption. A multilevel regression model will be applied to identify the organisational and individual determinants of EHR adoption in clinical settings. Alternative analytical models would be applied if necessary.

**Results:**

The study will assess the contribution of organisational and individual factors, as well as their interactions, to the implementation of EHR in clinical settings.

**Conclusions:**

These results will be very relevant for decision makers and managers who are facing the challenge of implementing EHR in the healthcare system. In addition, this research constitutes a major contribution to the field of knowledge transfer and implementation science.

## Background

Information and communication technologies (ICTs) include a set of effective tools to collect, store, process, and exchange health-related information [1]. In that respect, it is believed that ICT could improve safety, quality, and cost-efficiency of healthcare services. Among the applications of ICTs to the healthcare sector, the electronic health record (EHR) is viewed as the backbone supporting the integration of various tools (*e.g*., emergency information, test ordering, electronic prescription, decision-support systems, digital imagery, and telemedicine) that could improve the uptake of evidence into clinical decisions. Using such evidence in daily clinical practices could enable a safer and more efficient healthcare system [2,3].

Patients, professionals, organisations, and the public in general are thus expected to benefit from EHR implementation. International literature supports several benefits of EHRs for patients [4-11]. One of the main benefits reported is the increased quality of care resulting from patients having their essential health data accessible to their different providers [11,12]. Based on relevant disease management programs [10,13], EHR could support empowered citizens to actively take part in decisions regarding their health. The EHR is also a tool that facilitates knowledge exchange and decision making among healthcare professionals by providing them with relevant, timely, and up-to-date information [14-16].

### Current knowledge on EHR adoption

The implementation of EHR in healthcare systems is currently supported in many countries. In the US, the Institute of Medicine has qualified the EHR as 'an essential technology for healthcare' [17]. The development of a National Health Information Infrastructure (NHII) was then seen as the core for the implementation of EHR across the US [18]. However, the rate of EHR adoption by office physicians remains slow in this country [19]. The UK has launched its National Program for Information Technology (NPfIT), an initiative from the National Health Service (NHS) to move towards an electronic care record for patients and to connect general practitioner and hospitals. However, this strategy has not yet reached the expected adoption levels [20-23].

An increasing body of knowledge on EHR implementation shows that a majority of projects do not sustain over the experimentation phase [24,25]. Issues associated with the slow diffusion of the EHR include: important start-up investments, lack of financial incentives, uncertain payoffs, suboptimal technology, low priority, and resistance of potential users [26-28]. A comparative study of EHR adoption among general practitioners (GPs) in 10 countries showed that Canadian GPs ranked last [29]. Another study of EHR adoption by primary care physicians showed that only 23% of them were using the EHR in Canada, compared to 89% in the UK [30]. Also, perceptions towards the use of EHR may vary between health professionals groups, adding to the complexity of implementing this technology in a pluralist healthcare system [31]. Thus, understanding factors influencing EHR adoption is one of the key to ensure its optimal integration and, ultimately, benefits measurement within health system and population. Factors pertaining to users and their working environment have to be considered because many previous EHR projects have failed due to the lack of integration into practices and organisations [32,33].

Previous studies on factors affecting EHR adoption in healthcare settings have traditionally focused on a single aspect of this multidimensional phenomenon [31]. As such, studies have usually assessed the adoption determinants either at the organisational/systemic level or at the professional/individual level. With regard to individual factors, several studies on barriers and facilitators to physicians' EHR adoption have been conducted [34-37]. Other studies have explored factors associated with nurses' intention to adopt EHR [38,39]. Factors affecting the readiness of healthcare organisations to implement interoperable information systems have also been studied [40-42].

Other studies have explored EHR adoption determinants at different levels without considering their possible interdependence. For example, Simon *et al*. [19,25] have conducted a survey on EHR adoption by medical practices in Massachusetts exploring organisational, professional, and technological factors. Their results showed that larger practices (seven physicians or more), hospital-setting and teaching status were significant predictors of EHR adoption. However, EHR adoption by healthcare professionals working in a specific setting might be influenced by the characteristics of the organisation, which implies a hierarchical or clustered data structure.

In Quebec, Lapointe [31,43] conducted a multidimensional analysis on the adoption of hospital information system by nurses and physicians using a multiple case study. Her findings indicate that individual decision to adopt the system or not may conflict with the organisation's decision to implement this system. This study also supports the hypothesis that organisational, group, and individual factors all influence the adoption of information systems to various degrees. Nevertheless, to the best of our knowledge, possible interactions between factors influencing EHR adoption by specific groups of professionals at different levels have never been assessed quantitatively.

### Goal and objectives

Adoption of EHR by healthcare professionals is an essential condition to ensure that its expected benefits will materialise. However, there is a gap in knowledge regarding the specific influence of individual and organisational factors in determining EHR adoption. The aim of this study is thus to assess the unique contribution of individual and organisational factors on the adoption of EHR in healthcare settings, as well as possible interrelations between these factors.

Specifically, the study seeks to answer the following questions: which factors, at the individual and organisational levels (independent variables) predict EHR adoption by healthcare professionals (dependant variable)?; what are the unique contributions of individual and organisational factors in predicting EHR adoption?; and how are individual and organisational adoption factors interrelated?

### Theoretical frameworks of EHR adoption

The phenomenon of innovation is omnipresent in the healthcare system where new technologies and interventions are constantly introduced in order to improve the health of individuals and populations. Innovation can be studied at four distinct levels: the individual healthcare professionals; the healthcare professionals groups; the healthcare organisations; and the larger healthcare system [44]. Several theories can be used to explore the adoption of innovations at each of these levels. However, it is important to select theories according to a set of attributes, such as their predictive or explicative effectiveness and their ability to provide targets for intervention [45].

### Organisational factors

Many theoretical models have been used to investigate the organisational characteristics influencing technology adoption. Given the particular nature of healthcare organisations, Mintzberg's configuration theory [46] and the neo-institutional theory [47-49] propose relevant concepts to analyse the relationships between hospitals' characteristics and the adoption of information and communication technologies [31].

The organisational theoretical framework guiding this study results from literature reviews and empirical studies, coupled with the characteristics proposed in Mintzberg's configuration theory [46]. The structural components of the professional bureaucracy--the type of configuration usually found in healthcare organisations--are defined in Table 1. Concepts pertaining to the context in which a new technology is introduced, inspired by the neo-institutional theory [47,48], are also included in the framework. Furthermore, based upon results from previous studies [31,50-53], research hypotheses on the expected influence of each structural and contextual variable on EHR adoption are presented.

**Table 1:** Structural and contextual variables and their expected influence on EHR adoption

Variable	Description	Hypothesis
Horizontal specialisation	The division of work is negotiated between the various specialties rather than on a hierarchical basis.	1. Horizontal specialisation has a negative influence on EHR adoption.

Functional differentiation	Differentiation, *i.e*., how the work is divided, is based upon production units, or fields of expertise.	2. The influence of functional differentiation on EHR adoption depends on groups' values towards the system.

Decentralisation of power	Informal power is both vertically and horizontally decentralised. Power is dispersed towards the bottom of the hierarchical chain and professionals exert a control over decision processes.	3. Decentralisation of power has a variable influence on EHR adoption, depending on professionals' values towards the technology.

Size	Hospital size has usually been measured as the number of beds. In the case of other organisations, number of physicians.	4. Larger organisations are more likely to adopt EHR.

Competition	The number of hospitals in the health region.	5. Organisations in regions where there are other hospitals are more likely to adopt HER.

Localisation	Health care organisations in the Province of Quebec are located in urban, outlying, remote, or isolated regions.	6. Organisations located in remote and isolated regions are less likely to adopt EHR.

Teaching status	Organisations with a teaching status have a larger network because of the presence physicians and residents from university hospitals.	7. Organisations with a teaching status are more likely to adopt EHR.

### Individual factors

Several theoretical models can be applied to study EHR adoption by healthcare professionals. Most of them consist in frameworks developed in other scientific fields, such as psychology, education, and sociology. In this study, factors that are hypothesised to influence EHR adoption by individual healthcare professionals are borrowed from a set of validated theoretical frameworks.

### Diffusion of innovation

Among those frameworks, the Diffusion of Innovation (DOI) has received much attention in the study of ICT adoption in healthcare [54]. This model suggests that there are three main sources influencing the adoption and diffusion of an innovation, namely perceptions of innovation characteristics, characteristics of the adopter, and contextual factors [55]. This model has been applied to study the adoption of various information technologies in healthcare [39]. However, the DOI does not provide information on how to assess innovation characteristics. Furthermore, this model has been criticized for its lack of specificity [56].

### Technology acceptance model

The Technology Acceptance Model (TAM) [57] was specifically developed to understand user's acceptance of information technology. In its original version, the TAM is similar to the Theory of Reasoned Action [58], considering intention as the direct antecedent of behaviour, while attitude and social norms being the predictors of intention [57]. The particularity of the TAM is that it decomposes the attitudinal construct found in previous models into two distinct factors--perceived ease of use (PEU) and perceived usefulness (PU). However, the TAM has been simplified over time and the attitudinal and normative components have been dropped from the model, leaving PEU and PU as the sole predictors of intention [59]. Many studies have empirically tested the TAM for the prediction of adoption behaviours for various technologies, including healthcare professionals' acceptance of telemedicine [60,61] and computerized decision-support system [62].

The TAM was specifically developed in the field of ICT adoption and it proposes a set of constructs that can be measured among various groups of users [57]. One limitation of this model is that it does not consider the social environment in which the technology is introduced. Consequently, some authors have questioned its applicability to study healthcare professionals' behaviours [60]. Various efforts have been made to extend the TAM by either introducing variables from other theoretical models or by examining antecedents and moderators of perceived ease of use and perceived usefulness.

### Theories of reasoned action and planned behaviour

These two models are presented jointly because the Theory of Planned Behaviour (TPB) [63,64] constitutes an extension to the Theory of Reasoned Action (TRA) [58]. Both models were developed in the field of social psychology in order to understand a variety of human behaviours. The TRA [58] postulates that the realisation of a given behaviour (B) is predicted by the individual intention (I) to perform this behaviour. In turn, the individual intention is formed by two antecedents--attitude toward act or behaviour (AACT) and subjective norm (SN). AACT represents the evaluation of the advantages and disadvantages associated with the performance of a given behaviour, weighted by their relative importance. SN is the individual's perception that significant others will approve or disapprove the behaviour in question, weighted by individual's motivation to comply.

However, some behaviour might not be totally under volitional control, which means that they require specific resources, skills, or opportunities for an individual in order to perform them. Therefore, the TPB [63,64] proposes to add the perception of behavioural control (PBC)--the person's evaluation of the barriers related to the realisation of the behaviour and his or her perceived capacity to overcome them--as a direct determinant of the behaviour. Furthermore, the PBC can also act as an indirect determinant of the behaviour by influencing the intention. According to these models, the influence of external variables, such as age, gender, and personality traits, is usually mediated through theoretical constructs. Both the TRA and the TPB have shown good predictive validity to explain behaviour and behavioural intention [65]. Moreover, these theories have been successful in explaining different behaviours of healthcare professionals [66-70]. However, evidence shows that the correlation between behavioural intention and actual behaviour is usually small to moderate [65,71]. A meta-analysis of the intention-behaviour relation among healthcare professionals [72] has reported significant positive correlations between intention and self-reported behaviour. A recent systematic review of the application of social cognitive theories to understand healthcare professionals' intentions and behaviours also supports these models [70].

### Theory of interpersonal behaviour

Another model that has been used to understand acceptance behaviours with respect to ICT is the Theory of Interpersonal Behaviour (TIB) [73]. In essence, the TIB is similar to the other intention-behaviour models in that it also proposes a set of psychosocial factors that influence the realisation of a given behaviour. However, the TIB specifies that three direct determinants influence behaviour: intention, facilitating conditions, and habit. Intention refers to the individual's motivation regarding the performance of a given behaviour. Facilitating conditions represent perceived factors in the environment that can ease the realization of a given behaviour. Habit constitutes the level of 'routinisation' of a given behaviour, *i.e*., the frequency of its occurrence.

According to the TIB, the behavioural intention is formed by attitudinal normative beliefs. Attitudinal beliefs are formed by affective (affect) and cognitive (perceived consequences) dimensions. Affect represents an emotional state that the performance of a given behaviour evokes for an individual, whereas perceived consequences refer to the cognitive evaluation of the probable consequences of the behaviour. The TIB also incorporates two normative dimensions: social and personal norms. Social norms are composed by normative and role beliefs. Normative beliefs consist of the internalisation by an individual of referent people or groups' opinion about the realisation of the behaviour, whereas role beliefs reflect the extent to which an individual thinks someone of his or her age, gender and social position should or should not behave. The personal normative construct of the TIB is formed by personal normative belief, described as the feeling of personal obligation regarding the performance of a given behaviour, and self-identity, which refers to the degree of congruence between the individual's perception of self and the characteristics he or she associates with the realisation of the behaviour.

**Figure 1:**
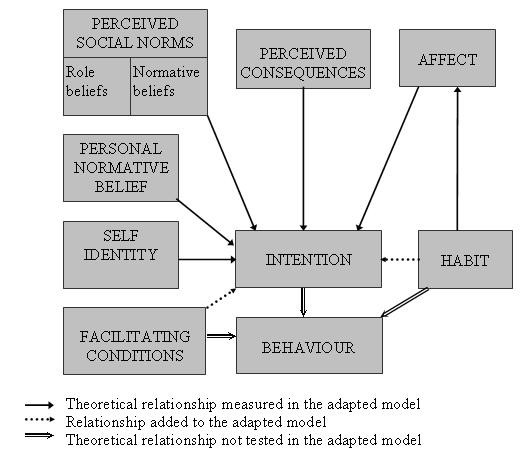
**Integrative theoretical framework to assess factors influencing EHR adoption at the individual level**. Adapted from the theory of Planned Behaviour [63] and the theory of Interpersonal Behaviour.

Compared to other intention-behaviour models, the TIB has a wider scope because it also considers cultural, social, and moral factors. The TIB was found to be a successful model to explain healthcare professionals' intention to perform clinical behaviours [70]. The TIB is also sensitive to cultural variations that affect the realisation of behaviours within specific social groups, such as healthcare professionals [74]. An integrative theoretical framework (Figure 1) will be used to assess factors influencing EHR adoption at the individual level based on the literature and previous research on healthcare professionals' behaviours conducted by the research team [66,67,75-77]. This framework comprises variables from the TPB and the TIB and has been applied in previous similar research [75,77].

## Methods

### Study design

A prospective cohort study will be used to identify the individual and organisational determinants of EHR adoption by healthcare professionals. This prospective design will follow study participants over time to verify how the determinants of EHR adoption evolve and to allow testing the predictive validity of the theoretical framework. Using Hierarchical Linear Model (HLM), the study will take into account the nested structure of data [78]. If no significant variation in the dependant variable (EHR adoption) is found across organisational units, then alternative analytical models would be applied.

### Population and settings

A stratified random sample of 50 healthcare organisations (HCOs) will be selected in the *Capitale Nationale *Health Region (Quebec City Health Region). This health region is divided into four Health and Social Services Centres (CSSS) that integrate a total of 78 units. The health region also includes 17 accredited Family Physicians Groups (FMGs). For the purpose of the study, a healthcare organisation is defined as a unit from one of the CSSS (including local community health centers, residential and long-term care centers, and hospital centers) or a FMG. HCOs targeted by the EHR project will be categorised in strata according to their size, mission, location, and nurses/physicians ratio. HCO in each stratum will be randomly ordered by an independent biostatistician. HCOs will be contacted and invited to participate in the study according to this random order until 60% of the HCO in each stratum have been recruited. If recruitment target of 60% is achieved in each stratum, a total of 50 HCO will be recruited. A sample of 50 clusters at the healthcare organisation level is usually considered as sufficient for longitudinal multilevel analyses [79].

In each HCO cluster, we aim to recruit a minimum of 15 and a maximum of 30 health professionals according to the size of the HCO. The sampling method will be similar to that used for HCO level. Potential participants in each HCO will be randomly stratified according to healthcare profession (physician and nurses). Recruitment will take into account the distribution of healthcare professionals in each HCO. We estimate a recruitment rate of 50% per HCO which corresponds to that of our preliminary work and to similar studies [25]. When the size of the units varies between organisations, it is suggested to calculate an average group size [80]. Our study sample will thus range between 750 and 1500 healthcare professionals which will be sufficiently powered to test the theoretical model of EHR adoption [81].

### Data collection instruments

#### Questionnaire for healthcare organisations

The HCO questionnaire measures structural and contextual organisational factors and is adapted from the literature [51,52] as well as on our previous work on telehealth adoption in HCO [82]. A preliminary version of this questionnaire was developed, and it will be face-validated by a convenient panel of five healthcare managers from the investigators' networks. This questionnaire will provide information about the organisational level factors that influence EHR adoption.

#### Questionnaire for healthcare professionals

Although adoption is considered as the key indicator of the success of EHR implementation by decision makers, no specific measure of this behaviour has been proposed [83,84]. It is thus important to provide a consensual measure of EHR adoption that can be used in the healthcare professionals' questionnaire. This cannot be achieved unless the behaviour is carefully defined in terms of its target, action, context, and time, which is known as the TACT approach [58]. Consequently, potential adoption behaviours identified from the literature on adoption and diffusion of innovations [54,85,86] will be classified for their relevance to the context of Quebec clinicians through a Delphi study among a panel of experts (see Foy and Bamford [87] for a similar procedure). The Delphi technique allows comparing the degree of written agreement among experts, and it is considered to be a strong methodology for a rigorous consensus of experts on a specific theme [88]. The results of the Delphi study will provide a consensus on the behaviours that will be used to calculate the composite adoption score in the healthcare professionals' questionnaire.

For the development of psychosocial questionnaires, Davidson *et al*. [89] recommend an etic-emic approach, inspired from the field of anthropology [90]. This method ensures the adaptation of theoretical concepts (the etic component) to the reality of the population under study (the emic component). This approach will be used to develop the questionnaire based on the theoretical constructs from the TIB [73] and the TPB [63,64]. To do so, two focus groups will be conducted among convenience samples of physicians and nurses. An experienced research professional trained in anthropology will moderate the focus groups. An open-ended guide will be used to assess participants' beliefs with respect to EHR adoption. Each question corresponds to a construct of the theoretical model. This questionnaire will assess psychosocial determinants of EHR adoption at the individual level and will be matched with HCO questionnaires.

### Data collection

At the organisational level, the HCO questionnaire will be administered by telephone at time I to two key informants, representing the managerial (the CEO or equivalent) and the professional (Director of Professional Services or equivalent) decision makers of each of the 50 organisations sampled. Key informants have been widely used in sociology, management, and marketing studies to obtain data on organisational variables [91,92]. Interviewing two respondents from each organisation will increase the convergent validity of data [93] and has been applied in a similar study [52]. The questionnaire will assess a set of structural and contextual characteristics from organisation theories. From our previous experience, we can expect a high response rate with this strategy (100% in our study of telehealth adoption [82]). Key informants from each participating organisations will be contacted again at time II, which will be between 12 and 18 months after the first data collection step, depending on the pace of EHR implementation in each organisation. The same questions will be used to monitor any important change in the organisation's structure or in its environment, and complementary questions will assess the organisation's progression towards EHR implementation.

At the individual level, individual questionnaires will be distributed at Time I to participating health professionals within each participating organisation. A study code will be assigned to each participant to facilitate follow up. The list of participants' names and codes will be kept confidential. A package containing a letter from the organisation's direction, a leaflet presenting the study, the study questionnaire, a consent form, and a reply envelope will be distributed to participants. At Time II (between 12 and 18 months, depending on the stage of EHR implementation), a second questionnaire will be distributed to the same participants to assess their current use of EHR. The second questionnaire will cover the same items as at Time I, but will also measure the frequency of use of the various components integrated in the EHR (*i.e*., laboratory tests, prescription database, digital imagery, and electronic clinical note). Because the sample is considered to be relatively stable, we do not anticipate major losses in follow-up. Our conservative sampling also secures a sufficient number of individual respondents by organisational units. Based on the specific adoption behaviours identified through the Delphi study, we will calculate a composite EHR adoption score by summing the score of each adoption behaviour measured, that will correspond to adoption patterns [52] or 'users trajectories' [94]. This categorical variable will be computed according to the trends observed in the global score of the adoption behaviours measured. For example, there could be three categories of adopters, corresponding to low, medium, and high adoption scores.

Furthermore, in order to account for bias inherent to self-reported measures, we will obtain objective utilisation data from the EHR system. Participants' consent will be sought to consult their utilisation of EHR components. The composite adoption score will thus be the dependant variable and we will assess which individual and organisational factors (independent variables) predict EHR adoption by healthcare professionals.

### Data analysis

Descriptive analyses of the data at each level (organisation and individual) will first be conducted to explore the distribution of socio-demographic and theoretical data. Statistics that are used to assess the reliability of individual data aggregated at group level in hierarchical models, such as the intra-class correlation (ICC1 and ICC2), the eta-squared (η^2^), and the omega-squared (ώ^2^) will be calculated. Then, the relevance of applying multilevel modelling to our data will be assessed by testing an unconditional or null model in which no predictors are specified. This allows verifying if significant variations in the dependant variable are present across healthcare organisations. If appropriate, a multilevel regression model [95] will be applied to identify organisational and individual determinants of EHR adoption in clinical settings. If no significant variation in EHR adoption is found across HCOs, a one-level path analysis model could be used [96]. If endogenous variables are normally distributed, Ordinary Least Squares (OLS) will be used. If, for specific equations, endogenous variables are not normally distributed, alternative non-linear models will be used. For all those analyses, we will use the MPLUS, version 5.21 [97]. This software allows conducting both path analysis and multilevel analysis with linear and non-linear data, and allows estimating specific indirect effects.

### Ethical considerations

The project has been approved by the ethics committee of the CHUQ Research Centre. Because the study population does not include patients, it is not required to seek ethics approval from other participating healthcare organisations. However, organisations solicited for participating in the project will be informed of the ethical aspects of the research and will receive copies of the research protocol and the ethics approval in order to ensure their informed decision to participate. The questionnaire for healthcare professionals will contain a unique code to identify study participants in order to facilitate follow-up. The list linking nominal information of participants to their study code will be kept in an electronic document protected by a password that will only be known by the principal investigator and the project coordinator. Other questionnaires and research materials will be anonymous.

## Discussion and implications

This study will provide unique knowledge on the most important factors to consider in the design of strategies for improving EHR adoption by healthcare professionals. As such, it will identify organisational and individual determinants that are key elements to the success of the ambitious interoperable EHR project promoted by the Canadian healthcare system. This project will be the first, to the best of our knowledge, to assess the unique contribution of organisational and individual factors, as well as their interactions, to the successful implementation of EHR. Moreover, the study will imply a wide range of healthcare settings to ensure greater generalisability of the results. These results will be particularly relevant and timely for decision makers who currently face the challenge of implementing EHR in the Canadian healthcare system. This study will apply a novel approach to assess adoption behaviour that is likely to be transferable to other settings. Furthermore, this research addresses some of the most important issues in the field of knowledge transfer and implementation science by proposing a theory-based, multilevel prospective longitudinal study that represents a major contribution to the field [98]. This project is also directly in line with current research priorities of the Canadian healthcare system identified by Listening for Direction III [99]. Finally, the project offers answers to priorities of the Canadian Institutes of Health Research Knowledge Synthesis and Exchange Branch because it will contribute to a better understanding of concepts, theories, and practices that underlie effective knowledge transfer in order to improve the health for Canadians, provide more effective health services and products, and strengthen the healthcare system.

## Competing interests

The authors declare that they have no competing interests.

## Authors' contributions

All authors collectively drafted the research protocol and approved the final manuscript. MPG is its guarantor.
